# FACILITY: feeding the family—the intergenerational approach to fight obesity, a cross-sectional study protocol

**DOI:** 10.3389/fped.2025.1450324

**Published:** 2025-03-31

**Authors:** Alessandra Vincenti, Valeria Calcaterra, Sara Santero, Giulia Viroli, Ilaria Di Napoli, Ginevra Biino, Luca Daconto, Mariaclaudia Cusumano, Gianvincenzo Zuccotti, Hellas Cena

**Affiliations:** ^1^ Laboratory of Dietetics and Clinical Nutrition, Department of Public Health, Experimental and Forensic Medicine, University of Pavia, Pavia, Italy; ^2^ Pediatric and Adolescent Unit, Department of Internal Medicine, University of Pavia, Pavia, Italy; ^3^ Pediatric Department, Buzzi Children’s Hospital, Milano, Italy; ^4^ Institute of Molecular Genetics, National Research Council of Italy, Pavia, Italy; ^5^ Department of Sociology and Social Research, University of Milano-Bicocca, Milan, Italy; ^6^ Department of Biomedical and Clinical Science, University of Milan, Milano, Italy; ^7^ Clinical Nutrition Unit, Department of General Medicine, ICS Maugeri IRCCS, Pavia, Italy

**Keywords:** paediatric obesity, healthy lifestyle, risk factors, mothers, environment, low socioeconomic status

## Abstract

**Introduction:**

Paediatric obesity has been described by the World Health Organization as one of the most serious health challenges of the 21st century. Over the past four decades, the number of children and adolescents with obesity has increased between 10 and 12-fold worldwide.

**Methods:**

Childhood obesity is a complex and multifactorial outcome which can be attributed to factors such as socioeconomic status, ethnicity, lifestyle and eating habits. Beside personal-children-related factors, maternal (education, food knowledge, income) and environmental ones (food environment's features and accessibility) have been proven but their influences are still worth discussion. The cross-sectional study of the “FACILITY: feeding the family—the intergenerational approach to fight obesity” project aims at estimating children prevalence of overweight and obesity and assessing the impacts of lifestyle and of socio-economic-cultural and environmental factors on overweight and obesity.

**Results:**

Due to the current importance of developing multidisciplinary mother-child centred prevention programs, FACILITY cross-sectional study will investigate maternal and child socio-cultural, economic, environmental, health and lifestyle-related risk factors for the development of obesity.

**Discussion:**

The knowledge gained will provide the basis to develop a “primordial prevention program” to early tackle childhood obesity.

**Clinical Trial Registration:**

ClinicalTrials.gov, identifier (NCT06179381).

## 1 Introduction

The Global Strategy for Women's, Children's, and Adolescents' Health (2016–2030) (1) plays a central role in the objective 3, Health and Well-being of the Agenda for Sustainable Development (2) for a future world in which every woman, child, and adolescent realises their rights to physical and mental health and well-being, in every setting (3). Obesity is defined as abnormal or excessive fat accumulation that poses a risk to health, due to an energy imbalance between energy intake and energy expenditure (4, 5). The reasons for this imbalance are complex and multifactorial (6). Health and nutritional status, lifestyle habits, biological features, environmental and socio-economic factors are nested within the family context, which in turn is nested within the community and the wider socio-geographical and cultural background (7).

Paediatric obesity has been described by the World Health Organization (WHO) as one of the most serious public health challenges of the 21st century (8). Over the past four decades, the number of children and adolescents with obesity has increased between 10 and 12-fold worldwide (9–11), with a rising prevalence among socially disadvantaged groups from low socio-economic backgrounds (11–13). In Italy, the prevalence of overweight and obesity for children aged 6–9 years is 29.8%, with the percentage of overweight and obesity around 20.4% and 9.4%, respectively (4).

Obesity prevention is recognized as the most feasible option for curbing the paediatric obesity epidemic (14). Most childhood obesity prevention programs have focused on school-aged children and have not proven so far to be effective in reversing the rising rates of childhood obesity (7). Indeed, by primary school entry, about 1 in 5 school-aged children were affected by obesity (15), suggesting that a prime opportunity to prevent childhood obesity has been missed (7). Thus, the prevention program should start very early, during and even before pregnancy and be shaped on mother-child dyads (16).

Maternal health is critical for healthy child growth and development (17). Indeed, maternal factors [i.e., Body Mass Index (BMI)], combined with an unhealthy family background (i.e., unhealthy eating habits and sedentary behaviour), strongly determine the lifestyle and nutrition behaviour of the child, predisposing him/her to the development of overweight or obesity (7, 18–20).

Along with the previously mentioned factors, socio-economic status (SES) has also been identified as a determinant for later obesity (11). Indeed, children with lower SES families have a steeper weight gain trajectory from birth with higher risk of obesity in adulthood (12, 21). Furthermore, children in poverty are more likely to become adults with lower SES and less wealth to pass on to future generations, in a vicious cycle, clearly acknowledging that obesity is increasingly related to poverty and is likely to be passed onto subsequent generations (22). Moreover, factors related to the socio-spatial environment play a key role in obesity (16, 23–25). For instance, the lack of healthy food availability, affordability and accessibility (e.g., food desert) has been positively linked to obesity. High neighbourhood walkability has been found to be associated with decreased prevalence of overweight and obesity, as well as the proximity to recreational facilities, parks, playgrounds, etc. have all been reported to be facilitators of physical activity (22, 26).

**Figure 1:**
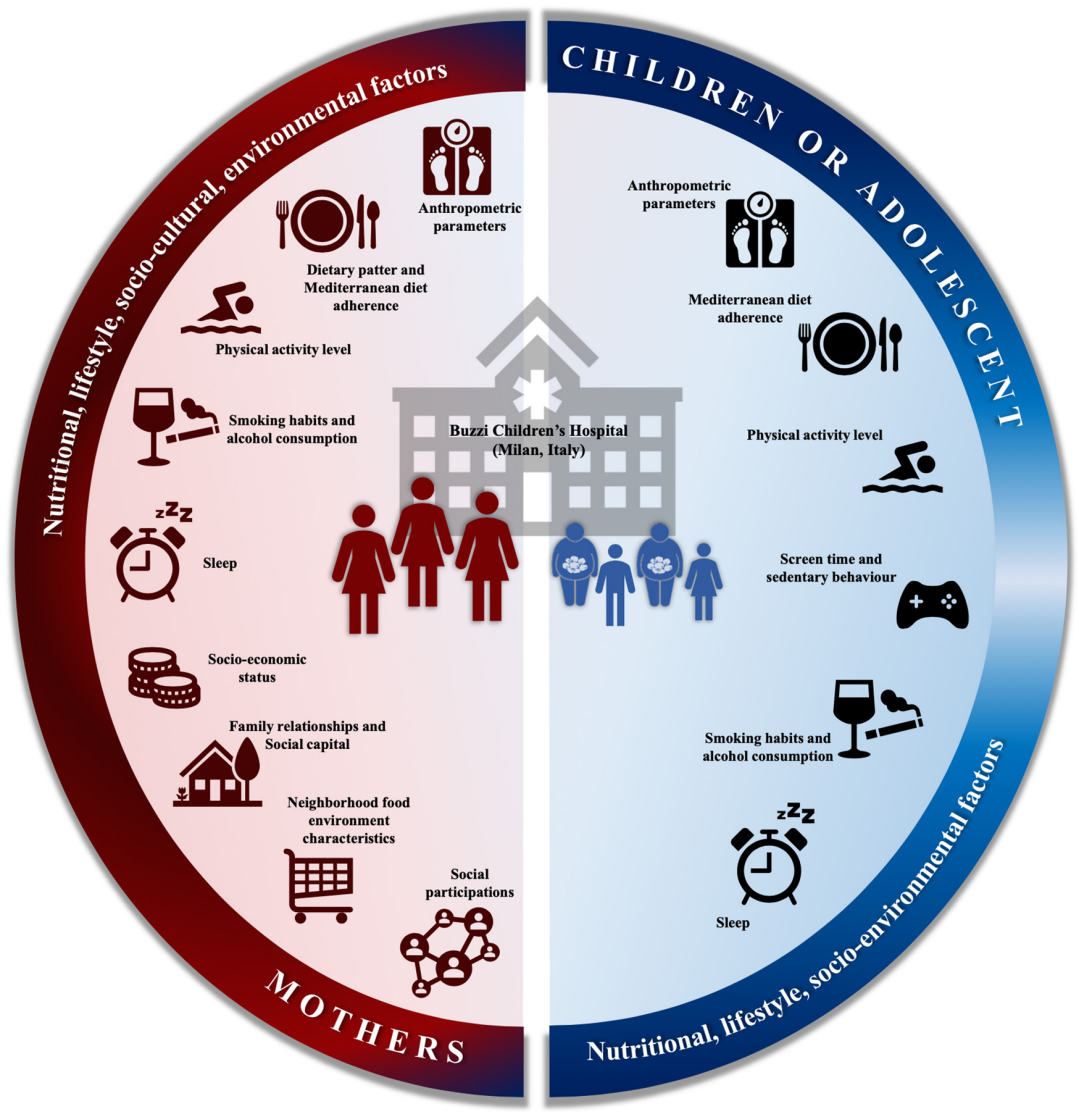
Graphical representation of FACILITY (feeding the family—the intergenerational approach to fight obesity) cross-sectional study. Understanding health and socio-economic-cultural maternal-child risk factors will enable the development of more effective public health policies for the prevention of childhood obesity to be drawn up in the future, according to the specific cultural and health needs of the target population.

The overall aim of the FACILITY (feeding the aamily—the intergenerational approach to fight obesity) project is to identify determinants for early onset of childhood obesity considering both maternal and children risk factors in a sample of mother-child dyads attending a paediatric outpatient clinic of the Buzzi Children's Hospital in Milan, Italy. More in detail, the cross-sectional study aims at (i) estimating the prevalence of overweight and obesity in the target population, thus, providing updated estimates in the Milan area, a useful data for the planning of public health interventions; (ii) assessing the impacts of lifestyle and of socio-economic-cultural and environmental factors on overweight and obesity (Figure 1).

## 2 Methods and analysis

### 2.1 Study setting

To address FACILITY's purposes, 269 mother-child dyads will be consecutively enrolled at the paediatric outpatient clinic of the Buzzi Children's Hospital in Milan, Italy. The enrollment will last from February 2024 until December 2025.

Eligible dyads according to the following inclusion and exclusion criteria will be involved in the study.

- Inclusion criteria:
  - For mothers: (i) age ≥18 years old, (ii) admission to the Department of Paediatrics, Buzzi Children's Hospital, (iii) ability to sign the informed consent.
  - For children/adolescents: (i) age between 2 and 18 years old, (ii) admission to the Department of Paediatrics, Buzzi Children's Hospital, (iii) informed consent signed by parents/local guardians.
- Exclusion criteria:
  - For mothers: (i) inability to understand the Italian and English language, (ii) refusal to sign the informed consent.
  - For children/adolescents: (i) presence of endocrine disorders (i.e., hypothyroidism, hypercortisolism, growth hormone deficiency), central nervous system damage (i.e., hypothalamic-pituitary damage because of surgery or trauma), genetic diseases either monogenic (i.e., leptin deficiency, MC4R mutation) or pleiotropic genetic syndromes (Prader-Willi, Bardet-Biedl), (ii) informed consent not written and signed by parents/local guardians.

Moreover, if mothers return to the paediatric outpatient clinic with another child, the latter will also be enrolled if the inclusion criteria of the study are met. Written informed consent will be obtained from the parent or legal guardians. Variables will be collected by a trained research team (clinicians, registered dietitians, paediatricians, biologists).

### 2.2 Assessed variables

A full list of the variables collected in the cross-sectional study is reported in Table 1. The clinical research team will collect the information of participants by consulting the patient's electronic/paper medical records and through validated questionnaires and structured interviews involving mothers at the enrollment. Structured interview characteristics are reported in Supplementary Table S1.

**Table 1:** List of variables collected in the cross-sectional study.

Variables	Methods	Sample
Demographic data	Structured interview	M
	Structured interview	C/A
Medical history and medication use	Structured interview	M
	Structured interview	C/A
Anthropometric parameters	Height, weight, BMI; waist circumference, direct measurement (27–29)	M
	Height, length or height; waist circumference, direct measurement (30, 31) Percentile of BMI, percentile of waist circumference, AR, calculated (30, 31)	C/A
Dietary pattern	Structured interview	M
Mediterranean Diet Adherence	Questionnaire MEDI LITE Score (32, 33)	M
	Questionnaire KIDMED (Diet Quality Index for Children and Adolescents) (34)	C/A
Physical activity level	International Physical Activity Questionnaire (IPAQ-SF; shortform) (35–37)	M
	Structured interview for children (38, 39)	C
	Questionnaire International Physical Activity Questionnaire for Adolescents (IPAQ-A) (40, 41)	A
Screen time and sedentary behaviour	Structured interview (38, 39)	C/A
Smoking habits and alcohol consumption	Structured interview (42)	M
	Structured interview (42)	A
Sleep	Questionnaire Pittsburgh Sleep Quality Index (PSQI) (43, 44)	M
	Structured interview (39, 45)	C/A
Socio-economic status	Calculated	M
Family relationships	Questionnaire Child-Parent relationship scale—Short Form (CPRS-SF) (46, 47)	M
Social capital	Questionnaire Personal Social Capital Scale (PSCS-8) (48)	M
Neighborhood food environment characteristics	Mapping and Spatial analysis; Structured interview	M
Social participations	Structured interview	M

M, mother; C, Children; A, Adolescent; BMI, Body Mass Index; AR, Adiposity Rebound.

#### Socio-demographic variables

Socio-demographic features will be collected with a structured interview. In detail, mother's age, marital status (marry/cohabitant, divorced/separated, widow, single), ethnicity (Caucasian, Black, Medioriental, Asiatic, Hispanic), nationality, education level (none, elementary, middle school, high school, degree), number of people in the household, number of children, number of years in Italy (if not Italian), residential address.

#### Economic, social, cultural and environmental variables

Socio-economic and socio-cultural characteristics will be collected through structured interviews. Information on household income, job/work and housing status (surface; owned, rented, sub-rented, social housing, guest, other) will be collected to detect socio-economic status.

Family relationships will be derived from the Child-Parent relationship scale-Short Form (CPRS-SF), a 15-item questionnaire assessing mothers' perception of their relationship with their son or daughter (46, 47). The Personal Social Capital Scale (PSCS-8) short form will be used to assess maternal durable and trustworthy social connections (48). Maternal social participation will be investigated through a structured interview to explore type (i.e., religious, recreational, cultural and, political) and frequency of activities.

Environmental factors that may influence nutrition, physical activity and lifestyle will be considered through the collection of geo-data on the characteristics of the food environment (e.g., the built environment, proximity to recreational/sport facilities, parks) and the transport infrastructure (e.g., walkability) (35, 49).

#### Medical history

Maternal and children/adolescent medical history will be explored through a structured interview (i.e., medical issues which includes all diseases and illnesses currently being treated and those which have had any residual effects on the mother and children/adolescent's health, medication history, surgical history and family medical history with potential indicators of predisposition to disease).

#### Anthropometric variables

Anthropometric measures of mothers will be collected using standardised procedures (27–29), weight (kg) will be measured with a calibrated weighing scale wearing underpants only (accuracy ± 100 g), height (cm) by a fixed stadiometer with a vertical backboard and a moveable headboard (accuracy ± 1 mm) and waist circumference (cm) will be measured with an elastic tape.

Height (cm) and weight (kg) will be collected also for children/adolescents. Briefly, standing height will be measured as previously described and according to standard procedures (28, 29). Recumbent length will be assessed for children who still are unable to stand up (29). Body weight of unclothed children/adolescents will be collected using a balanced weight scale (accuracy ± 100 g), following standardised procedures (29). WHO child growth standards will be used to diagnose overweight (BMI Z-score >1) or obesity (BMI Z-score ≥2) (50). Infant growth curves from the paediatric booklet will be requested to evaluate the time (year) of the adiposity rebound. The presence of an early adiposity rebound (EAR) will be identified when the lowest BMI during child growth occurs at an age <5 years old (30, 31) and children/adolescents will be categorised into early or non-early adiposity rebound (AR). Waist circumference (cm) will be collected in children/adolescents using standardised procedures (29).

#### Lifestyle variables

##### Mediterranean dietary pattern adherence, self-perception of the current diet and eating attitude

For mothers, Mediterranean dietary adherence will be explored using the 9-item MEDI-LITE questionnaire (32, 33). The questionnaire investigates the frequency of consumption of nine classes of food (i) fruit, (ii) vegetables, (iii) cereal grains, (iv) legumes, (v) fish and fish products, (vi) meat and meat products, (vii) dairy products, (viii) alcohol intake, and (ix) olive oil. The score obtained from the questionnaire ranges from 0 to 18, where the highest value corresponds to the highest MD adherence (33). Moreover, a structured interview will be also used to evaluate self-perception of the current diet, to investigate any dietary restrictions (i.e., carbohydrates, fat, meat, fish, eggs, dairy, gluten, lactose) or consumption of traditional food/dishes.

For children, Mediterranean dietary adherence will be assessed with the KIDMED (Diet Quality Index for Children and Adolescents) questionnaire (34). The KIDMED is a 16-item tool used for the evaluation of consumption of fruit, fruit juice, vegetables, fish, pulses, cereals, nuts, olive oil and yoghurts or cheese. KIDMED also explores fast-foods, commercial bakery goods and pastries, sweets and candy consumption, as well as eating behaviours (i.e., skipping breakfast). In addition, for children, a structured interview of two items will assess the endorsement of any of the following binge eating symptoms (i) sneaking, hiding, or hoarding food, (ii) eating in the absence of hunger. These questions have been selected in previous trials with the aim to examine the prevalence of overeating symptoms in overweight or obesity affected children and adolescents (51–53).

#### Physical activity

Maternal physical activity will be assessed by the short form (7 items) of a validated country-language-specific questionnaire [International Physical Activity Questionnaire (IPAQ—SF)] (36). The questionnaire provides an estimate of the metabolic equivalent of task (MET-min) per week, which is calculated as follows: METs = MET level ×  minutes of activity × events per week. Physical activity level will be classified according to METs into sedentary (total METs <699), moderate (total METs between 700 and 2,519), and high (total METs >2,520) (37, 54).

For children aged 2–14 years old, physical activity habits will be explored through a structured interview, differentiating questions according to the different age groups (3–4 years, 5–14 years) as suggested by WHO guidelines for physical activity (38, 39).

For adolescents aged 15–17 years old, the Italian version of the International Physical Activity Questionnaire for Adolescents (IPAQ-A) will be used to investigate time spent in physical activities in different settings (i) school-related physical activity, (ii) housework, house maintenance and gardening, (iii) transportation, (iv) recreation, sport, and leisure-time physical activity (40, 41). The quantity of physical activity will be calculated by multiplying the energy expenditure required (MET) and the amount of time spent (min) in a week for each activity. Moderate intensity will be weighed in absolute terms as 4 METs, vigorous intensity defined as 8 METs and walking as 3.3 MET.

Children's sedentary screen time behaviours will be also assessed through a structured interview, differentiating the use of screens (i.e., TV, other devices such as smartphone, computer, tablet, touch screen, and game console) at home and school for both weekdays and weekends. Total screen exposure results will be compared to WHO guidelines (38, 39).

#### Alcohol consumption and smoking habit

For mothers and adolescents (12–18 years old) interviews will be used to assess smoking habits and alcohol intake. Concerning smoking habits usage of traditional tobacco cigarettes (manufactured or hand-rolled cigarettes), electronic cigarettes or heated tobacco products (HTP) will be evaluated. Participants will be then categorised as follows (i) never smoker, (ii) past smoker (i.e., current non-smoker), (iii) current smoker.

According to the frequency of alcohol intake, the respondents will be segmented in three categories (i) abstainers [i.e., subjects who do not drink alcohol, zero Units of Alcohol (U.A.)], (ii) occasional drinkers (i.e., subjects who drink less than 1 U.A./week), (iii) daily drinkers (i.e., subjects who regularly drink alcohol, more than 1 U.A./week). According to the Italian “Linee Guida per una sana alimentazione” (42) 1 U.A. equals 12 g of pure alcohol contained in a small glass of red/white/rosé wine (125 ml, alcohol by volume 12%), one can of double malt beer (330 ml, ABV 4.6%), or a small shot of hard liquor (40 ml, ABV 40%).

#### Sleep

Pittsburgh Sleep Quality Index (PSQI) (43, 44) will be used to explore sleep quality of mothers. The PSQI is a 9-item questionnaire assessing 7 sub-dimensions of sleep (i.e., subjective sleep quality, sleep latency, sleep duration, habitual sleep efficiency, sleep disturbances, use of sleeping medication and daytime dysfunction).

For children, sleep quality will be assessed through a structured interview regarding sleep and wake-up times, sleep regularity, average number of hours of sleep per night, difficulty falling asleep and daytime sleepiness. Results will be compared to WHO guidelines (39, 45).

### 2.3 Data collection and management

Participants' data will be collected in both electronic and paper form and sent by authorised study personnel to an electronic database protected by a password. Each individual participant and their data will be identified with a unique study identification number (pseudo-anonymization of data), which will prevent any direct identification. The unique identifier will be used from that point forward on all relevant documentation. The Coordinator Centre (Laboratory of Dietetics and Clinical Nutrition) will be responsible for the data storage in a locked and secured location. Laboratory of Dietetics and Clinical Nutrition and Buzzi Children's Hospital will be responsible for data entry and quality.

### 2.4 Data analysis

In the cross-sectional study, sample size has been determined considering a prevalence of overweight and obesity in the target population of 17% and 4%, respectively (55). Considering a 95% level of confidence and 5% precision, 217 individuals for overweight and 52 for obesity prevalence estimates are required, for a total sample size of 269 mother-child dyads.

Enrolled subjects will be described with reference to all the collected variables by means of standard descriptive statistics. Prevalence of overweight and obesity will be estimated along with 95% confidence intervals. In order to assess the impacts of lifestyle and socio-economic, cultural, and environmental factors on overweight and obesity, logistic regression will be adopted. Overweight and obesity in children, as binary variables, will be the response in two separate logistic models, where socio-economic, cultural, and environmental factors, among all collected data, will be the independent variables. Indeed, performing different analyses according to the BMI status (overweight/obesity) will capture different health correlations, since the greater severity of overweight and obesity usually corresponds to an unhealthy lifestyle, and to a low SES (12, 21). Analyses will also be adjusted for potential confounders like age, sex or any other variable identifiable as such among the collected data.

Furthermore, if sample size allows, subgroups analysis by ethnicity (Caucasian or non-Caucasian) will be performed since it likely results in different cultural habits, impacting nutrition and other lifestyle factors (56, 57).

Missing data will be handled with multiple imputation.

## 3 Discussion

The cross-sectional study of the FACILITY project aimed at evaluating potential risk factors associated with the onset of childhood obesity through a comprehensive analysis of maternal and children/adolescent health, lifestyle, economic, social and environmental variables.

Obesity is defined by WHO as a complex disease with physical, social and psychological dimensions, resulting in serious health and economic consequences (5, 8). Previous studies have shown positive associations between unhealthy child lifestyle, such as adherence to a Western dietary pattern, poor sleep and sedentary behaviour, and obesity development (18). However, excessive weight gain occurs across the life cycle. Indeed, children born from mothers who are overweight or obese at the time of conception have a higher risk of obesity compared to children born from mothers with normal weight, creating intergenerational cycles of obesity (12, 18, 22, 58). Family-based behavioural treatments have been identified as one of the main interventions for an effective childhood obesity prevention and treatment, underlying the importance of the family habits for the shape of child's lifestyle behaviour (59). In addition, environmental and maternal cultural influences on child's weight are unique topics, which are only recently investigated (12, 60–62). However, scientific literature often provides partial and sometimes contradictory findings regarding the association of many of the individual and socio-cultural factors on obesity (63–67). Therefore, new researches that combine different factors are needed, with a focus on family lifestyle, health, environment and socio-cultural variables (56). In this scenario, the FACILITY project aims to provide evidence on the association between individual, family and socio-economic and cultural factors, and excessive body weight during childhood and adolescence. Thus, the study may fill the gap between the interplay of social-cultural and health domains in the Italian context, since to the authors' knowledge no studies on maternal and infant socio-economic variables have been published in Italy (68–73). The cross-sectional study of the FACILITY project has numerous strengths. First, it evaluates a broad range of risk factors across socio-economic, cultural, health, and lifestyle dimensions. Second, the age range is broad (i.e., 2–18), potentially allowing the assessment of all-age children and adolescents. Often, studies focus on one specific age class, therefore the first exploratory analysis will capture the differences in risk factors manifestations. Third, it will use visual inspection of BMI-for-age plots, which is the gold standard for defining timing of AR. Also, maternal and infant anthropometric variables will be measured by qualified medical professionals.

Nevertheless, the cross-sectional phase of the FACILITY project has few limitations. First, due to the observational study design of the two study phases, a direct cause-and-effect relationship between the examined variables and overweight, obesity, and EAR can not be ascribed. Second, FACILITY focuses on mother-child dyads, excluding fathers from the analysis, although several evidence is highlighting the role of the triads for the child's development into adulthood (74, 75). Third, due to the specificity of the setting (single outpatient clinic in a North-Italian hospital), the results obtained from this study will not be generalizable to the entire Italian population. While children attending the outpatient clinic may have different health conditions and socio-economic backgrounds, it should be noted that visits are offered free of charge, which limits the participation of families with higher incomes who might prefer private healthcare settings, potentially leading to the underrepresentation of these groups. Moreover, the study will be conducted at a specific clinic, which may not reflect the diversity of healthcare settings across different regions of Italy, thus reducing the generalizability of the results to the national level.

Besides, the sampling frame may introduce a bias due to the non-random method of participant selection. Since children will be consecutively enrolled at the outpatient clinic, the sample may not be fully representative of the broader pediatric population. The recruitment method could result in a non-homogeneous distribution of age groups, as some age ranges may be more likely to attend the clinic due to specific health concerns.

Fourth, some of the outcome measures rely on parental reports and the use of structured interviews may lead to interviewer bias. Whenever possible, the authors have selected validated questionnaires, however, to date no validated tools to capture the mother's diet and her cultural influences exist. Therefore, structured interviews have been developed by the research team, which may mean that real lifestyle habits of the mothers and children would not be properly assessed due to the lack of validation of the interviews. Fifth, the authors recognize the lack of evaluation of additional potentially interesting variables correlated to childhood obesity (e.g., body composition, metabolic syndrome, food insecurity, screen time during mealtimes, family meals or shared meals).

In conclusion, the cross-sectional study of the FACILITY project aims to shed light on the intricate web of lifestyle-related, social, economic, cultural, and environmental risk factors contributing to the early onset of obesity during childhood and adolescence within mother-child dyads. The study stands poised to offer updated, comprehensive data and a potential blueprint for action to Italian policymakers and healthcare professionals, with the goal of mitigating the childhood obesity epidemic. Given the elevated incidence of obesity and unwholesome habits among vulnerable demographics, particularly women and children from underprivileged backgrounds, it becomes imperative to tailor childhood obesity prevention policies to their needs, thereby addressing the decrement in the age of obesity onset. It is also critical to adapt current Caucasian-centric prevention strategic policies to more effectively serve the Italian population, ensuring they consider the diverse social, economic, and cultural factors that influence the health of future generations. This study underscores the importance of extending the focus beyond traditional approaches, advocating for policies that reflect the nuances of ethnic diversity and their bearing on child health outcomes.

The FACILITY project encapsulates an articulate response to an urgent public health matter, proffering a multi-dimensional analysis whose extrapolation could lead to transformative public health solutions. It robustly adheres to a methodological framework capable of shedding lights on complex interactions, and the need for expanding its demographic inclusivity and enriching the dataset with information about paternal influence is admitted in the study. If carefully followed up on, these projects will improve our knowledge of the phenomena and create an even more effective approach to addressing childhood obesity. Consequently, the project anticipates offering invaluable lessons on the intergenerational dynamics at play, fostering an evidence-based foundation upon which to construct more culturally responsive and equitable public health policies.

## 4 Ethics and dissemination

The FACILITY study was approved by the Ethics Committee (Comitato Etico Territoriale Lombardia 1) on 6 November 2023 (approval code: CET 120-2023, version 3, December 06, 2023). The study will be conducted according to the Declaration of Helsinki guidelines, to International Guidelines and to current Clinical Trial laws.

The study protocol was registered in ClinicalTrials.gov (ID: NCT06179381). If modifications in the original protocol will be required, additional amendments will be demanded to the Ethic Committee. In this scenario, the approved related information will be updated on clinicaltrial.gov.

To ensure a greater scientific rigour, the authors followed the SPIRIT and STROBE-NUTR guidelines for study protocols reporting (76, 77).

The study design and results will be publicly available at the OnFoods website (www.onfoods.it, info@onfoods.it) in line with the transparency policy. The results of the FACILITY study will be published in indexed scientific journals, regardless of whether they are positive, negative, or inconclusive at the end of the study. Participants' data will be published anonymously.
