# Supplementary material for: FACILITY: feeding the family—the intergenerational approach to fight obesity, a cross-sectional study protocol
Source: Front Pediatr. 2025 Mar 31;13:1450324. doi: 10.3389/fped.2025.1450324 (PMC11994582; doi:10.3389/fped.2025.1450324)
Supplement: Supplementary file 1 [file Datasheet1.docx]

***Supplementary Material***

# Supplementary Material 1 - Checklist: SPIRIT checklist;

SPIRIT 2013 Checklist: Recommended items to address in a clinical trial protocol and related documents*

| **Section/item** | **Item N°** | **Description** | **Section** |
| --- | --- | --- | --- |
| **Administrative information** | | |  |
| Title | 1 | Descriptive title identifying the study design, population, interventions, and, if applicable, trial acronym | Title |
| Trial registration | 2a | Trial identifier and registry name. If not yet registered, name of intended registry | Abstract |
|  | 2b | All items from the World Health Organization Trial Registration Data Set | - |
| Protocol version | 3 | Date and version identifier | Ethics and dissemination |
| Funding | 4 | Sources and types of financial, material, and other support | Funding |
| Roles and responsibilities | 5a | Names, affiliations, and roles of protocol contributors | Abstract |
|  | 5b | Name and contact information for the trial sponsor | Ethics and dissemination |
|  | 5c | Role of study sponsor and funders, if any, in study design; collection, management, analysis, and interpretation of data; writing of the report; and the decision to submit the report for publication, including whether they will have ultimate authority over any of these activities | - |
|  | 5d | Composition, roles, and responsibilities of the coordinating centre, steering committee, endpoint adjudication committee, data management team, and other individuals or groups overseeing the trial, if applicable (see Item 21a for data monitoring committee) | - |
| **Introduction** |  |  |  |
| Background and rationale | 6a | Description of research question and justification for undertaking the trial, including summary of relevant studies (published and unpublished) examining benefits and harms for each intervention | Introduction |
|  | 6b | Explanation for choice of comparators | no because it is a cross-sectional |
| Objectives | 7 | Specific objectives or hypotheses | Introduction |
| Trial design | 8 | Description of trial design including type of trial (eg, parallel group, crossover, factorial, single group), allocation ratio, and framework (eg, superiority, equivalence, noninferiority, exploratory) | Introduction |
| **Methods: Participants, interventions, and outcomes** | | |  |
| Study setting | 9 | Description of study settings (eg, community clinic, academic hospital) and list of countries where data will be collected. Reference to where list of study sites can be obtained | Methods and analysis, Study setting |
| Eligibility criteria | 10 | Inclusion and exclusion criteria for participants. If applicable, eligibility criteria for study centres and individuals who will perform the interventions (eg, surgeons, psychotherapists) | Methods and analysis, Study setting |
| Interventions | 11a | Interventions for each group with sufficient detail to allow replication, including how and when they will be administered | it is an observational study |
|  | 11b | Criteria for discontinuing or modifying allocated interventions for a given trial participant (eg, drug dose change in response to harms, participant request, or improving/worsening disease) | it is an observational study |
|  | 11c | Strategies to improve adherence to intervention protocols, and any procedures for monitoring adherence (eg, drug tablet return, laboratory tests) | it is an observational study |
|  | 11d | Relevant concomitant care and interventions that are permitted or prohibited during the trial | it is an observational study |
| Outcomes | 12 | Primary, secondary, and other outcomes, including the specific measurement variable (eg, systolic blood pressure), analysis metric (eg, change from baseline, final value, time to event), method of aggregation (eg, median, proportion), and time point for each outcome. Explanation of the clinical relevance of chosen efficacy and harm outcomes is strongly recommended | Methods and analysis, Study setting, Assessed variables  Table 1 and Supplementary materials |
| Participant timeline | 13 | Time schedule of enrolment, interventions (including any run-ins and washouts), assessments, and visits for participants. A schematic diagram is highly recommended (see Figure) | Methods and analysis, Assessed variables |
| Sample size | 14 | Estimated number of participants needed to achieve study objectives and how it was determined, including clinical and statistical assumptions supporting any sample size calculations | Data analysis |
| Recruitment | 15 | Strategies for achieving adequate participant enrolment to reach target sample size | Methods and analysis, Study setting. Data analysis |
| **Methods: Assignment of interventions (for controlled trials)** | | |  |
| Allocation: |  |  |  |
| Sequence generation | 16a | Method of generating the allocation sequence (eg, computer-generated random numbers), and list of any factors for stratification. To reduce predictability of a random sequence, details of any planned restriction (eg, blocking) should be provided in a separate document that is unavailable to those who enrol participants or assign interventions | it is an observational study |
| Allocation concealment mechanism | 16b | Mechanism of implementing the allocation sequence (eg, central telephone; sequentially numbered, opaque, sealed envelopes), describing any steps to conceal the sequence until interventions are assigned | it is an observational study |
| Implementation | 16c | Who will generate the allocation sequence, who will enrol participants, and who will assign participants to interventions | it is an observational study |
| Blinding (masking) | 17a | Who will be blinded after assignment to interventions (eg, trial participants, care providers, outcome assessors, data analysts), and how | it is an observational study |
|  | 17b | If blinded, circumstances under which unblinding is permissible, and procedure for revealing a participant’s allocated intervention during the trial | it is an observational study |
| **Methods: Data collection, management, and analysis** | | |  |
| Data collection methods | 18a | Plans for assessment and collection of outcome, baseline, and other trial data, including any related processes to promote data quality (eg, duplicate measurements, training of assessors) and a description of study instruments (eg, questionnaires, laboratory tests) along with their reliability and validity, if known. Reference to where data collection forms can be found, if not in the protocol | Methods and analysis, Assessed variables. Data analysis  Supplementary materials |
|  | 18b | Plans to promote participant retention and complete follow-up, including list of any outcome data to be collected for participants who discontinue or deviate from intervention protocols | it is an observational study |
| Data management | 19 | Plans for data entry, coding, security, and storage, including any related processes to promote data quality (eg, double data entry; range checks for data values). Reference to where details of data management procedures can be found, if not in the protocol | Data analysis |
| Statistical methods | 20a | Statistical methods for analysing primary and secondary outcomes. Reference to where other details of the statistical analysis plan can be found, if not in the protocol | Data analysis |
|  | 20b | Methods for any additional analyses (eg, subgroup and adjusted analyses) | Data analysis |
|  | 20c | Definition of analysis population relating to protocol non-adherence (eg, as randomised analysis), and any statistical methods to handle missing data (eg, multiple imputation) | Data analysis |
| **Methods: Monitoring** | | |  |
| Data monitoring | 21a | Composition of data monitoring committee (DMC); summary of its role and reporting structure; statement of whether it is independent from the sponsor and competing interests; and reference to where further details about its charter can be found, if not in the protocol. Alternatively, an explanation of why a DMC is not needed | - |
|  | 21b | Description of any interim analyses and stopping guidelines, including who will have access to these interim results and make the final decision to terminate the trial | it is an observational study |
| Harms | 22 | Plans for collecting, assessing, reporting, and managing solicited and spontaneously reported adverse events and other unintended effects of trial interventions or trial conduct | it is an observational study |
| Auditing | 23 | Frequency and procedures for auditing trial conduct, if any, and whether the process will be independent from investigators and the sponsor | - |
| **Ethics and dissemination** | | |  |
| Research ethics approval | 24 | Plans for seeking research ethics committee/institutional review board (REC/IRB) approval | Ethics and dissemination |
| Protocol amendments | 25 | Plans for communicating important protocol modifications (eg, changes to eligibility criteria, outcomes, analyses) to relevant parties (eg, investigators, REC/IRBs, trial participants, trial registries, journals, regulators) | Ethics and dissemination |
| Consent or assent | 26a | Who will obtain informed consent or assent from potential trial participants or authorised surrogates, and how (see Item 32) | Methods and analysis, study setting Supplementary material |
|  | 26b | Additional consent provisions for collection and use of participant data and biological specimens in ancillary studies, if applicable | - |
| Confidentiality | 27 | How personal information about potential and enrolled participants will be collected, shared, and maintained in order to protect confidentiality before, during, and after the trial | Data analysis |
| Declaration of interests | 28 | Financial and other competing interests for principal investigators for the overall trial and each study site | Funding |
| Access to data | 29 | Statement of who will have access to the final trial dataset, and disclosure of contractual agreements that limit such access for investigators | Data analysis. Conflict of interest |
| Ancillary and post-trial care | 30 | Provisions, if any, for ancillary and post-trial care, and for compensation to those who suffer harm from trial participation | - |
| Dissemination policy | 31a | Plans for investigators and sponsor to communicate trial results to participants, healthcare professionals, the public, and other relevant groups (eg, via publication, reporting in results databases, or other data sharing arrangements), including any publication restrictions | Ethics and dissemination |
|  | 31b | Authorship eligibility guidelines and any intended use of professional writers | - |
|  | 31c | Plans, if any, for granting public access to the full protocol, participant-level dataset, and statistical code | - |
| **Appendices** |  |  |  |
| Informed consent materials | 32 | Model consent form and other related documentation given to participants and authorised surrogates | Supplementary material |
| Biological specimens | 33 | Plans for collection, laboratory evaluation, and storage of biological specimens for genetic or molecular analysis in the current trial and for future use in ancillary studies, if applicable | - |

*It is strongly recommended that this checklist be read in conjunction with the SPIRIT 2013 Explanation & Elaboration for important clarification on the items. Amendments to the protocol should be tracked and dated. The SPIRIT checklist is copyrighted by the SPIRIT Group under the Creative Commons “[Attribution-NonCommercial-NoDerivs 3.0 Unported](http://www.creativecommons.org/licenses/by-nc-nd/3.0/)” license.

# Supplementary Material 2 – Checklist: STROBE-nut

**STROBE-nut: An extension of the STROBE statement for nutritional epidemiology**

Lachat C et al. (2016) STrengthening the Reporting of OBservational studies in Epidemiology – Nutritional Epidemiology (STROBE-nut): an extension of the STROBE statement. Plos Medicine 13(6) <http://dx.doi.org/10.1371/journal.pmed.1002036> [pdf](http://journals.plos.org/plosmedicine/article/asset?id=10.1371%2Fjournal.pmed.1002036.PDF) or [online](http://journals.plos.org/plosmedicine/article?id=10.1371/journal.pmed.1002036) version.

| **Item** | **Item nr** | **STROBE recommendations** | **Extension for Nutritional Epidemiology studies (STROBE-nut)** | **Reported on page #** |
| --- | --- | --- | --- | --- |
| **Title and**  **abstract** | 1 | (a) Indicate the study’s design with a commonly used term in the title or the abstract.  (b) Provide in the abstract an informative and balanced summary of what was done and what was found. | **nut-1** State the dietary/nutritional assessment method(s) used in the title, abstract, or keywords. | **Title.**  **Abstract**  **Nut-1: mentioned in other parts of the manuscript** |
| **Introduction** |  |  |  |  |
| Background rationale | 2 | Explain the scientific background and rationale for the investigation being reported. |  | **Introduction** |
| Objectives | 3 | State specific objectives, including any pre-specified hypotheses. |  | **Introduction** |
| **Methods** |  |  |  |  |
| Study design | 4 | Present key elements of study design early in the paper. |  | **Methods and analysis, study setting** |
| Settings | 5 | Describe the setting, locations, and relevant dates, including periods of recruitment, exposure, follow-up, and data collection. | **nut-5** Describe any characteristics of the study settings that might affect the dietary intake or nutritional status of the participants, if applicable. | **Methods and analysis, study setting** |
| Participants | 6 | a) Cohort study—Give the eligibility criteria, and the sources and methods of selection of participants. Describe methods of follow-up.  Case-control study—Give the eligibility criteria, and the sources and methods of case ascertainment and control selection. Give the rationale for the choice of cases and controls.  Cross-sectional study—Give the eligibility criteria, and the sources and methods of selection of participants.  (b) Cohort study—For matched studies, give matching criteria and number of exposed and unexposed.  Case-control study—For matched studies, give matching criteria and the number of controls per case. | **nut-6** Report particular dietary, physiological or nutritional characteristics that were considered when selecting the target population. | **Methods and analysis, study setting** |
| Variables | 7 | Clearly define all outcomes, exposures, predictors, potential confounders, and effect modifiers. Give diagnostic criteria, if applicable. | **nut-7.1** Clearly define foods, food groups, nutrients, or other food components.  **nut-7.2** When using dietary patterns or indices, describe the methods to obtain them and their nutritional properties. | **Methods and analysis, study setting. Assessed variables**  **(nut-7.1;nut-7.2)**  **Supplementary material** |
| Data sources - measurements | 8 | For each variable of interest, give sources of data and details of methods of assessment (measurement).Describe comparability of assessment methods if there is more than one group. | **nut-8.1** Describe the dietary assessment method(s), e.g., portion size estimation, number of days and items recorded, how it was developed and administered, and how quality was assured. Report if and how supplement intake was assessed.  **nut-8.2** Describe and justify food composition data used. Explain the procedure to match food composition with consumption data. Describe the use of conversion factors, if applicable.  **nut-8.3** Describe the nutrient requirements, recommendations, or dietary guidelines and the evaluation approach used to compare intake with the dietary reference values, if applicable.  **nut-8.4** When using nutritional biomarkers, additionally use the STROBE Extension for Molecular Epidemiology (STROBE-ME). Report the type of biomarkers used and their usefulness as dietary exposure markers.  **nut-8.5** Describe the assessment of nondietary data (e.g., nutritional status and influencing factors) and timing of the assessment of these variables in relation to dietary assessment.  **nut-8.6** Report on the validity of the dietary or nutritional assessment methods and any internal or external validation used in the study, if applicable. | **Assessed variables Table 1**  **Supplementary material** |
| Bias | 9 | Describe any efforts to address potential sources of bias. | **nut-9** Report how bias in dietary or nutritional assessment was addressed, e.g., misreporting, changes in habits as a result of being measured, or data imputation from other sources | **Data analysis** |
| Study Size | 10 | Explain how the study size was arrived at. |  | **Data analysis** |
| Quantitative variables | 11 | Explain how quantitative variables were handled in the analyses. If applicable, describe which groupings were chosen and why. | **nut-11** Explain categorization of dietary/nutritional data (e.g., use of N-tiles and handling of nonconsumers) and the choice of reference category, if applicable. | **Assessed variables** |
| Statistical  Methods | 12 | (a) Describe all statistical methods, including those used to control for confounding  (b) Describe any methods used to examine subgroups and interactions.  (c) Explain how missing data were addressed.  (d) Cohort study—If applicable, explain how loss to follow-up was addressed.  Case-control study—If applicable, explain how matching of cases and controls was addressed.  Cross-sectional study—If applicable, describe analytical methods taking account of sampling strategy.  (e) Describe any sensitivity analyses. | **nut-12.1** Describe any statistical method used to combine dietary or nutritional data, if applicable.  **nut-12.2** Describe and justify the method for energy adjustments, intake modeling, and use of weighting factors, if applicable.  **nut-12.3** Report any adjustments for measurement error, i.e,. from a validity or calibration study. | **245-259** |
| **Results** |  |  |  |  |
| Participants | 13 | (a) Report the numbers of individuals at each stage of the study—e.g., numbers potentially eligible, examined for eligibility, confirmed eligible, included in the study, completing follow-up, and analyzed.  (b) Give reasons for non-participation at each stage.  (c) Consider use of a flow diagram. | **nut-13** Report the number of individuals excluded based on missing, incomplete or implausible dietary/nutritional data. | **It is a study protocol** |
| Descriptive data | 14 | (a) Give characteristics of study participants (e.g., demographic, clinical, social) and information on exposures and potential confounders  (b) Indicate the number of participants with missing data for each variable of interest  (c) Cohort study—Summarize follow-up time (e.g., average and total amount) | **nut-14** Give the distribution of participant characteristics across the exposure variables if applicable. Specify if food consumption of total population or consumers only were used to obtain results. | **It is a study protocol** |
| Outcome data | 15 | Cohort study—Report numbers of outcome events or summary measures over time.  Case-control study—Report numbers in each exposure category, or summary measures of exposure.  Cross-sectional study—Report numbers of outcome events or summary measures. |  | **It is a study protocol** |
| Main results | 16 | (a) Give unadjusted estimates and, if applicable, confounder-adjusted estimates and their precision (e.g., 95% confidence interval).  Make clear which confounders were adjusted for and why they were included.  (b) Report category boundaries when continuous variables were categorized.  (c) If relevant, consider translating estimates of relative risk into absolute risk for a meaningful time period. | **nut-16** Specify if nutrient intakes are reported with or without inclusion of dietary supplement intake, if applicable. | **It is a study protocol** |
| Other analyses | 17 | Report other analyses done—e.g., analyses of subgroups and interactions and sensitivity analyses. | **nut-17** Report any sensitivity analysis (e.g., exclusion of misreporters or outliers) and data imputation, if applicable. | **It is a study protocol** |
| **Discussion** |  |  |  |  |
| Key results | 18 | Summarize key results with reference to study objectives. |  | **It is a study protocol** |
| Limitation | 19 | Discuss limitations of the study, taking into account sources of potential bias or imprecision. Discuss both direction and magnitude of any potential bias. | **nut-19** Describe the main limitations of the data sources and assessment methods used and implications for the interpretation of the findings. | **Discussion (it is a study protocol)** |
| Interpretation | 20 | Give a cautious overall interpretation of results considering objectives, limitations, multiplicity of analyses, results from similar studies, and other relevant evidence. | **nut-20** Report the nutritional relevance of the findings, given the complexity of diet or nutrition as an exposure. | **It is a study protocol** |
| Generalizability | 21 | Discuss the generalizability (external validity) of the study results. |  | **It is a study protocol** |
| **Other information** |  |  |  |  |
| *Funding* | 22 | Give the source of funding and the role of the funders for the present study and, if applicable, for the original study on which the present article is based. |  | **Funding** |
| *Ethics* |  |  | **nut-22.1** Describe the procedure for consent and study approval from ethics committee(s). | **Ethics and dissemination** |
| *Supplementary material* |  |  | **nut-22.2** Provide data collection tools and data as online material or explain how they can be accessed. | **Supplementary material** |

# Supplementary Material 3 - Structured interviews

For the mother

- Dietary pattern of the mother
- Neighborhood food environment characteristic
- Social participation

For the child/adolescent

- Physical activity, screen time and sedentary behaviour
- Sleep habits
- Binge eating symptoms

| **DIETARY PATTERN OF THE MOTHER** | |
| --- | --- |
| *In this section the mother will be asked for some information about her current dietary habits.* | |
| **How do you consider your current diet?**  *(Healthy: oil as a source of fat, rich in fruits, vegetables, whole grains, legumes, nuts; moderate consumption of animal products; occasional consumption of processed meat and sweets.*  *Western: high consumption of refined flours, meat and processed meat, fried and precooked foods, sugary non-alcoholic  drinks, butter... )* | - Healthy and balanced - Unhealthy (western diet) |
| **Do you apply any particular restrictions?** | Yes  No |
| **(If yes) Which one?** | - Carbohydrates - Fat - All meat types (meat and fish) - Only some types of meat: _____________ - Fish - Eggs - Cheese - Gluten - Lactose - Other _________________ |
| **For which reason(s)?** | - Ethical - Religious - Health-related - Economic - Other |
| **Do you eat food or recipes typical of your tradition/country of origin?** | - Yes - No |
| **If yes, which one (s)?** | _____________________________________ |
| **Do you habitually follow food traditions typical of your country of origin?** *(e.g. drinking the in the afternoon)* | - Yes - No |
| **If yes, which one(s)?** | _____________________________________ |
| **Has living in Italy influenced your eating habits?** | - Yes - No |
| **(If yes), which eating habits?** | _____________________________________ |

| **NEIGHBOURHOOD FOOD ENVIRONMENT CHARACTERISTIC** | |
| --- | --- |
| *In this section the mother’s perception of the food environment surrounding her house will be explored.* | |
| How much do you agree with the following statement about your neighbourhood? | |
| **There are many grocery shops** | - Completely agree - Quite agree - Neither agree nor disagree - Quite disagree - Completely disagree |
| **There is a large foods variety in the grocery shops** | - Completely agree - Quite agree - Neither agree nor disagree - Quite disagree - Completely disagree |
| **Food products are cheap** | - Completely agree - Quite agree - Neither agree nor disagree - Quite disagree - Completely disagree |
| **The quality of the foods products is high** | - Completely agree - Quite agree - Neither agree nor disagree - Quite disagree - Completely disagree |

| **SOCIAL PARTICIPATION** | |
| --- | --- |
| *In this section the type and frequency of activities in which the mother takes part will be explored* | |
| **Cultural (cinema, library, theater ...)** | - More than once a week - Once a week - Once every 15 days - Once every month - Never |
| **Religious (parish ...)** | - More than once a week - Once a week - Once every 15 days - Once every month - Never |
| **Recreational (going to the park, going out with family and/or friends, playing ...)** | - More than once a week - Once a week - Once every 15 days - Once every month - Never |
| **Politics (unions, parties, committees ...)** | - More than once a week - Once a week - Once every 15 days - Once every month - Never |

| **PHYSICAL ACTIVITY, SCREEN TIME AND SEDENTARY BEHAVIOUR** | |
| --- | --- |
| *In this section the child’s physical activity, screen time and sedentary behaviour will be explored .*  Q*uestions will be asked  to the mother only if her child is < 12 years old.*  *In the case of adolescents (12-18 years old), questions will be asked directly to the participant.*  *Only for physical activity, International Physical Activity Questionnaire (IPAQ) will be provided to participants from 14 to 18 years old.*  *It is necessary to fill in only the section corresponding to the age of the child.* | |
| ***Physical activity*** | |
| **2 years old**  **How much movement** **does your child do during the day?** *(e.g., playing outoors, riding a bike, chasing games and ball games)* | - <30 min - 30 min - 1 hour - 1  hour 1/2 - 2  hours - 2  hours 1/2 - 3  hours - > 3  hours |
| **3-4 years old**  **How much movement (light intensity activities) does your child do during the day?** *(e.g., slow walking, bathing)*  *Please* *sum different moments of the day*  **How long is moderate-vigorous intensity physical activity carried out during the day?** *(e.g.,* *brisk walking, cycling, running playing ball games, swimming, dancing)*  *Please sum different moments of the day* | - <30 min - 30 min - 1 hour - 1  hour 1/2 - 2  hours - 2  hours 1/2 - 3  hours - > 3  hours |
|  | - <30 min - 30 min - 1 hour - 1  hour 1/2 - 2  hours - 2  hours 1/2 - 3  hours - > 3  hours |
| **5-14 years old**  **How long moderate-vigorous physical activity is carried out per day on weekdays?** *(predominantly aerobic activities: e.g., brisk walking, cycling, running playing ball games, swimming, dancing)*  **How long moderate-vigorous physical activity is carried out per day on weekends?** *(predominantly aerobic activities)*    **How long vigorous intensity physical activity is carried out per week?** *(aerobic and muscle strengthening)* | - <30 min - 30 min - 1 hour - 1  hour 1/2 - 2  hours - 2  hours 1/2 - 3  hours - > 3  hours |
|  | - <30 min - 30 min - 1 hour - 1  hour 1/2 - 2  hours - 2  hours 1/2 - 3  hours - > 3  hours |
|  | - <30 min - 30 min - 1 hour - 1  hour 1/2 - 2  hours - 2  hours 1/2 - 3  hours - > 3  hours |
| ***Sedentary behaviour*** | |
| **2-4 years old**  **How long do you leave your child in the pram/stroller/mother's back or continuously in a sitting position ?** | - 30 min - 1 hour - 1  hour 1/2 - 2  hours - 2  hours 1/2 - 3  hours - > 3  hours |
| ***Screen time*** | |
| **All ages**  **How much time do your child / do you spend daily in front of the computer in your free time on school days? (including video games)** | - Never - 30 min - 1 hour - 2  hours - 3  hours - 4 hours - 5 or more hours |
| **How much time do your child / do you spend daily in front of the computer in your free time on the weekend? (including video games)** | - Never - 30 min - 1 hour - 2  hours - 3  hours - 4 hours - 5 or more hours |
| **How much time do your child / do you spend daily watching television/movies in your free time on school days? (including video games)** | - Never - 30 min - 1 hour - 2  hours - 3  hours - 4 hours - 5 or more hours |
| **How much time do your child / do you spend daily watching television/movies in your free time on the weekend? (including video games)** | - Never - 30 min - 1 hour - 2  hours - 3  hours - 4 hours - 5 or more hours |
| **How much time do your child / do you spend daily on your cell phone in your free time on school days?** | - Never - 30 min - 1 hour - 2  hours - 3  hours - 4 hours - 5 or more hours |
| **How much time do your child / do you spend daily on your cell phone overall in your free time on the weekend?** | - Never - 30 min - 1 hour - 2  hours - 3  hours - 4 hours - 5 or more hours |

| **SLEEP HABITS** | |
| --- | --- |
| *In this section the child’s sleep habits will be explored.* Q*uestions will be addressed  to the mother only if her child is < 12 years old.*  *In the case of adolescents (12-18 years old), questions will be assessed directly to the participant.* | |
| **What time did your child / did you go to bed last night ?** |  |
| **What time did your child / did you wake up this morning?** |  |
| **Do your child / do you follow this time schedule regularly?** | - Yes   Specified ________________   - No |
| **On average, how many hours do your child / do you sleep per night?** | _________________________________________ |
| **Do your child / do you have difficulty falling asleep?** | - Yes - No |
| **Do your child / do you have daytime sleepiness?** | - Yes - No |

| **BINGE EATING SYMPTOMS** | |
| --- | --- |
| *In this section the presence of binge eating symptoms (BES) will be explored. This questionnaire does not provide a diagnosis but it evaluates the presence of early signs of disruptured eating behaviors in the child.* | |
| *Questions addressed to the mother if the child is <12 years old* | |
| **Do you think your child is secretly sneaking, hiding or hoarding food?** | - Yes - No |
| **Do you think your child feels inhibited or embarrassed when eating in front of others compared to when eating alone?** | - Yes - No |
| *Questions addressed to the child if the age is ≥12 years old* | |
| **Do you tend to secretly sneak/hide or hoard food?** | - Yes - No |
| **Do you feel inhibited/embarrassed when eating in front of others compared to when eating alone?** | - Yes - No |
